# Future Acceptability of Respiratory Virus Infection Control Interventions in General Population to Prevent Respiratory Infections

**DOI:** 10.3390/medicina58070838

**Published:** 2022-06-22

**Authors:** Jaber S. Alqahtani, Abdulelah M. Aldhahir, Saad M. AlRabeeah, Lujain B. Alsenani, Haifa M. Alsharif, Amani Y. Alshehri, Mayadah M. Alenazi, Musallam Alnasser, Ahmed S. Alqahtani, Ibrahim A. AlDraiwiesh, Saeed M. Alghamdi, Rayan A. Siraj, Hussain S. Alqahtani, Jithin K. Sreedharan, Abdullah S. Alqahtani, Eidan M. Alzahrani

**Affiliations:** 1 Department of Respiratory Care, Prince Sultan Military College of Health Sciences, Dammam 34313, Saudi Arabia; lujainalsenani@gmail.com (L.B.A.); alsharif.haifa1@gmail.com (H.M.A.); amaniyahay98@gmail.com (A.Y.A.); mayadahalenezi@gmail.com (M.M.A.); musalllam@gmail.com (M.A.); ahmed@psmchs.edu.sa (A.S.A.); ialdraiwiesh@gmail.com (I.A.A.); jithinksree@gmail.com (J.K.S.); rt0075@hotmail.com (A.S.A.); 2 Respiratory Therapy Department, Faculty of Applied Medical Sciences, Jazan University, Jazan 45142, Saudi Arabia; aldhahir.abdulelah@hotmail.com; 3 Clinical Technology Department, Respiratory Care Program, College of Applied Medical Sciences, Umm Al-Qura University, Makkah 21961, Saudi Arabia; s.alghamdi18@imperial.ac.uk; 4 National Heart and Lung Institute, Imperial College London, London SW7 2BX, UK; 5 Respiratory Therapy Department, King Faisal University, Al-Ahsa 31982, Saudi Arabia; rayan_as18@hotmail.com; 6 Department of Clinical Laboratory Sciences, Prince Sultan Military College of Health Sciences, Dammam 34313, Saudi Arabia; halqahtani@psmchs.edu.sa; 7 Physical Therapy Department, Prince Sultan Military College of Health Sciences, Dammam 34313, Saudi Arabia; edan@psmchs.edu.sa

**Keywords:** respiratory infections, COVID, public health, social distance, smoking

## Abstract

*Background and Objectives*: In both pandemic and non-pandemic situations, nonpharmaceutical public health measures may offer easy, low-cost, and effective means of reducing the spread and impact of acute respiratory infections. It is unknown whether such measures would be acceptable to the Saudi community beyond the current pandemic. *Materials and Methods*: A validated survey was used to test community acceptance of the measures. Respondents were asked which infection control practices they planned to maintain and which they believed should be policies for the community as a whole after the COVID-19 pandemic has subsided. *Results:* The survey was completed by 2057 people (95% completion rate), 1486 (72%) of whom were female, 259 (12.5%) of whom were current smokers, and 72 (3.5%) of whom had chronic lung disease. The most prevalent age groups were 18–30 years (933; 45.4%) and 31–40 years (483; 23.5%), with 641 individuals over 40 years old. Of the responses, 93% indicated that they would continue washing their hands more often; 92% wanted both clinicians and patients to wear masks in hospitals; 86% would continue avoiding smoking in indoor and outdoor areas; 73% would continue wearing a face covering on public transportation; 70% indicated that they would continue wearing a face covering in indoor public places. Regarding the respiratory virus infection control measures, 85% (11/13) received significant support (≥70% acceptability level) for continuation as policies in the future. Wearing face coverings outdoors and social distancing outdoors received little support (45% and 66%, respectively). Of the respiratory virus infection control measures, 54% received less support from current smokers than non-smokers (acceptability level < 70%). People with chronic respiratory disease supported 77% of the measures being regarded as policies in the future. *Conclusion:* The Saudi community supports nonpharmacological respiratory infection control measures that reduce the likelihood of infection. Public health campaigns should target smokers to increase awareness of the importance of these measures in lowering infections. Based on the findings of this study, nonpharmacological treatments should be presented and included in future recommendations for both the public and patients diagnosed with chronic respiratory diseases.

## 1. Introduction

The wide spread of acute respiratory infections, particularly the severe acute respiratory syndrome coronavirus-2 (SARS-CoV-2), has raised immense global public health concerns. The increasing number of infection rates due to SARS-CoV-2 led the World Health Organization (WHO) to announce a state of international emergency and the start of the SARS-CoV-2 pandemic in 2020 [1]. Ever since, global health authorities have increased their efforts to reduce the transmission of viral infections and minimize the growing burden introduced by the SARS-CoV-2 pandemic.

SARS-CoV-2 is a highly contagious pathogen that requires robust surveillance and control measures [2,3]. Respiratory infection control incorporates pharmaceuticals, such as vaccinations and nonpharmaceutical interventions (NPIs), including indoor or outdoor social distancing, hand hygiene, and mask-wearing [4]. However, since the mass production of vaccinations can be challenging, especially in low-income and developing countries [5], the use of NPIs has been considered the primary protection strategy against the SARS-CoV-2 virus [6,7].

NPIs can provide effective, convenient, and affordable techniques for mitigating the rapid spread of acute respiratory infections during pandemic and non-pandemic times [8,9]. Results from a recent systematic review have shown that the efficacy of NPIs targeting SARS-CoV-2 has gone beyond expectations and reduced the rate of influenza virus infections [10]. Moreover, the use of NPIs was also associated with an apparent reduction in the number of hospital admissions for patients living with chronic airway diseases during the SARS-CoV-2 pandemic [11,12]. This was confirmed by another recent systematic review that showed a 50% reduction in hospitalization rate for patients suffering from chronic obstructive pulmonary disease (COPD) exacerbation during the SARS-CoV-2 pandemic when compared to pre-pandemic levels, most likely due to NPI use [13]. However, this finding may be explained by the fact that vulnerable people may take extra preventive measures to avoid contracting SARS-CoV-2 [14]. Ultimately, these results may have encouraged several global health authorities to mandate a strict use of NPIs during the SARS-CoV-2 pandemic. However, the effectiveness of NPIs still relies mainly on perception, knowledge, adherence, and acceptance by the public [4].

Although there is growing evidence supporting public knowledge and compliance with NPIs during the current SARS-CoV-2 pandemic and previous viral epidemics, studies exploring the acceptability rate for the future use of NPIs by the general population are still lacking [15,16,17]. Only one study has assessed the long-term acceptability of NPIs to prevent future exacerbations in people living with chronic airway diseases [18]. This online large-scale cross-sectional survey found that 79.5% of the respondents would continue washing their hands, and 68.6% would keep social distancing indoors [18]. These findings recommend measuring the acceptance and adoption levels of NPIs by the general population in the future, particularly once the SARS-CoV-2 pandemic has subsided. This might be the first step in developing international guidelines and, additionally, informing health officials regarding public readiness to mitigate any potential upcoming SARS-CoV-2 variants or other future respiratory pandemics.

This study aimed to evaluate the level of public acceptance toward maintaining the same infection control practices used during the SARS-CoV-2 pandemic and whether the public feels the need for formal health policies and legislation mandating the use of NPIs to prevent future respiratory infections. The results will also contribute to gaining a better understanding of the public beliefs and perceptions regarding infection preventive measures, particularly once SARS-CoV-2 restrictions have been eased.

## 2. Materials and Methods

This research used a modified version of a previously validated assessment survey [18]. The survey is adaptive and consists of three sections. Section one covers general information regarding demographic data, education level, region, presence of comorbidities, smoking history, history of SARS-CoV-2 infection, and vaccination status. Section 2 includes five questions about the respondents’ beliefs about COVID-19. Section 3 asks participants their opinions about nonpharmacological interventions to reduce respiratory virus transmission, which includes 13 measures. All of these questions were purposefully constructed to span a period after the COVID-19 pandemic has ended. In addition, the survey asks participants for their recommendations regarding which measures should be in effect for the general population at all times.

In Saudi Arabia, Arabic is the official language and the primary language of communication for the majority of the people. The survey’s original form was written in English, therefore it was handed to the Professional Translation Unit at Prince Sultan Military College of Health Sciences to translate it into the Arabic language. The Arabic version was then translated back into English by another professional translation expert. Both expert translators in the Translation Unit used “forward–backward translation” to compare the two English versions as recommended by the World Health Organization. A pilot test was then performed on 15 people from the public who fulfilled the inclusion criteria for the study, utilizing the Arabic version of the validated questionnaire. Participated people reported that the questionnaire was simple to understand and complete.

The survey was hosted online using Google Forms, and the link to the survey was openly available on social media platforms to all residents of Saudi Arabia across all regions, who older than 18 years old. The research team also approached the public in malls and shopping centers to increase the response rate. A paper-based survey was offered to those who lacked digital literacy and was later manually entered by the research team. The aims of the research, the estimated time for completing the survey, data confidentiality, and the fact that participation was optional were all explained to the participants. No monetary or nonmonetary incentives were presented. Before submitting the survey, the respondents were permitted to examine all of their replies. The use of IP addresses ensured that there was no duplication of answers. The Institutional Review Board of Prince Sultan Military College of Health Sciences, Saudi Arabia, granted ethical approval. The completion of the survey was regarded as an agreement to participate. Personal information was anonymized and destroyed as soon as it was processed.

### Data Analysis

The data were compiled in Excel and analyzed using SPSS (version 28). A Chi-squared analysis was used to compare proportions between groups. The statistical significance level was set at *p* < 0.05. A ≥70% cut-off of responses was arbitrarily and a priori selected as showing broad support for a measure, while <70% support was considered a lack of substantial support. A difference of more than 10 percentage points between groups was chosen arbitrarily and a priori to suggest a potentially important difference.

## 3. Results

The study was completed by 2057 people (95% completion rate), including 1486 (72%) females, and 1114 (54.2%) participants with a bachelor’s degree. The majority (835; 40.6%) were from the western region of Saudi Arabia. The most common age categories were 18–30 years (933; 45.4%) and 31–40 years (483; 23.5%), with 641 participants older than 40 years (Table 1). Of the participants, 1812 (88.1%) were from urban areas, and the common comorbidities reported among respondents were high blood pressure (128; 6.2%), diabetes mellitus (123; 6%), anemia (85; 4.1%), stomach disease (81; 3.9%), and chronic lung disease (72; 3.5%).

The smoking history was as follows: 1697 (82.5%) had never smoked, 259 (12.5%) were current smokers, and 101 (4.9%) were former smokers. More than half of the current smokers had attempted to quit during the COVID-19 pandemic (174/259 = 67%). Of the respondents, 889 (43.2%) had a previous COVID-19 infection, and the majority had mild (250; 28%) to moderate severity (501; 56%). Almost all the respondents (2028; 98%) were vaccinated against COVID-19, and 1202 (59%) of the respondents had a booster dose.

### 3.1. Beliefs about COVID-19

We asked participants about their beliefs about COVID-19. Most respondents (1942; 94%) believed that COVID-19 is spreading fast. They also believed it is media hyped (1456; 71%), something they worry about all the time (1436; 70%), stressful (1389; 68%), and fear-inducing (973; 47%).

### 3.2. Future Acceptability with No Policies

Table 2 shows the survey responses to the longer-term acceptability of the respiratory virus infection control measures. Nine out of the 13 respiratory virus infection control interventions (69%) had an acceptability level of 70% or more among the survey respondents. A significant number of respondents predicted that they would continue to take precautionary measures to avoid respiratory infections in the future. The top five infection control measures people would continue in the future were washing their hands more often (1910; 93%), wearing masks in hospital settings (1886; 92%), self-isolating if they were in contact with someone who was infected with COVID-19 (1878; 91%), being vaccinated (1793; 87%), and avoiding smoking in indoor and outdoor areas (1759; 86%). There was less support (acceptability level < 70%) for wearing face coverings outdoors, social distancing outdoors, and avoiding seeing friends or family if they were unwell with a cold or flu, and hand sanitizer being widely available to clean one’s hands.

**Figure 1:**
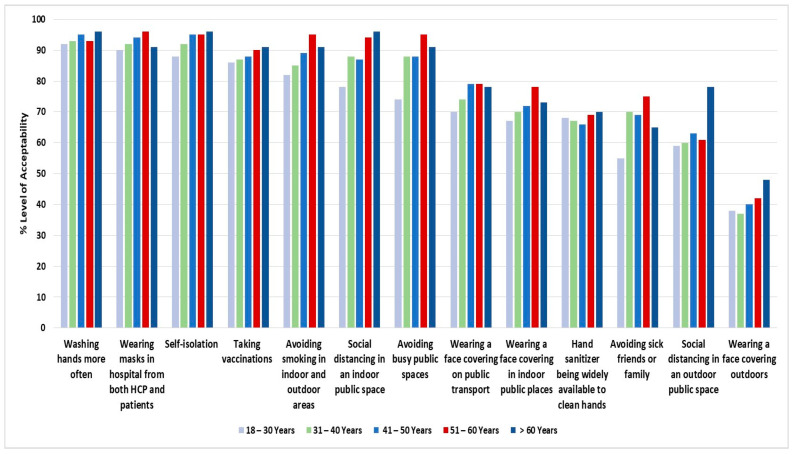
The acceptability of policies regarding respiratory virus infection control interventions by age.

When stratified by age groups, there were statistically significant differences, and differences of more than 10%, between groups regarding wearing a face covering in indoor public places (*p* value = 0.03), wearing a face covering on public transportation (*p* value = 0.002), maintaining social distancing from others when in an indoor public space (*p* value < 0.001), avoiding busy public spaces (*p* value < 0.001), avoiding seeing friends or family if they were unwell with a cold or flu (*p* value < 0.001), avoiding smoking in indoor and outdoor areas (*p* value < 0.001), both health-care providers (HCP) and patients wearing masks in hospitals (*p* value = 0.009), and self-isolating if they were in contact with someone who was infected with COVID-19 (*p* value < 0.001). Figure 1 shows the variation in the acceptability levels stratified by age group.

### 3.3. Acceptability of Future Policies

**Figure 2:**
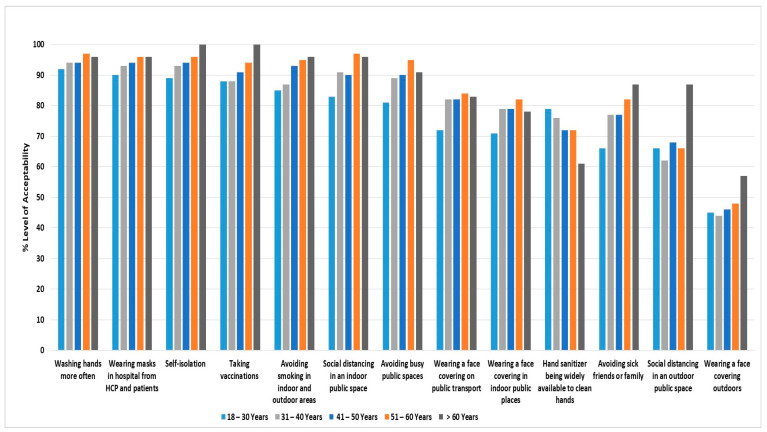
The acceptability of future policies for everyone at all times regarding respiratory virus infection control interventions by age.

Of the respiratory virus infection control measures, 85% received great support (11/13), defined as ≥ 70% acceptability level, for continuation in the future as policies for everyone at all times. Only wearing face coverings outdoors and social distancing outdoors received little support as future policies for everyone (45 and 66%, respectively). Figure 2 presents the level of future policy acceptability for each respiratory virus infection control measure, stratified by age group. Hand sanitizer availability to clean hands was less supported by those older than 60 years (*p* value = 0.01) when compared to other age groups. Likewise, avoiding visiting friends or family if they have a cold or flu was less supported by young people when compared to other groups (*p* value < 0.001). Social distancing in outdoor public spaces was only supported by those who were older than 60 years (acceptability level was 87%), although there was no statistical significance among groups (*p* value = 0.09). Wearing a face covering outdoors was less supported by all age groups (acceptability level < 70%).

**Figure 3:**
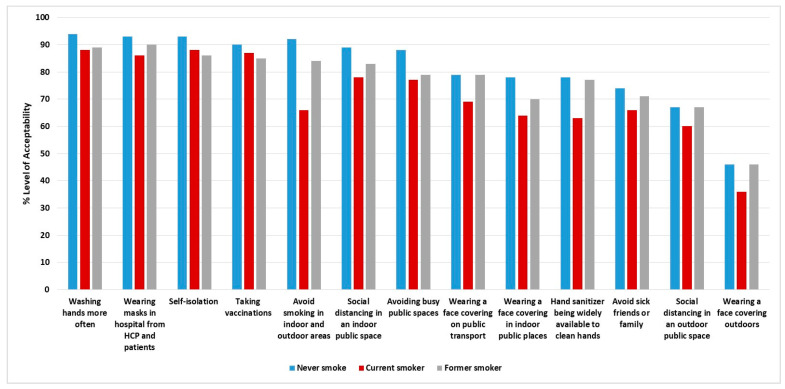
The acceptability of future policies for everyone at all times regarding respiratory virus infection control interventions by smoking status.

**Figure 4:**
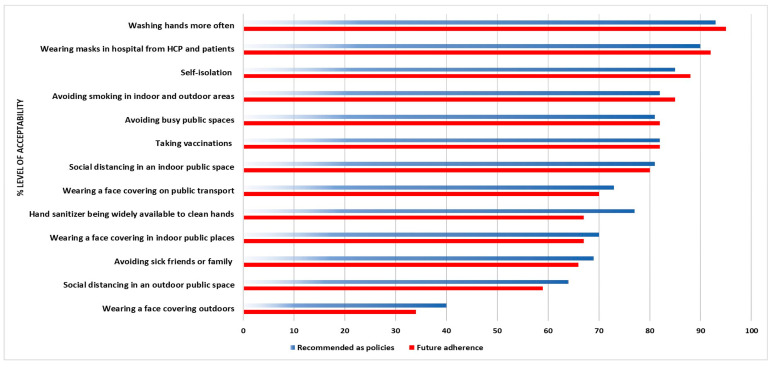
The acceptability of respiratory virus infection control interventions in people with chronic respiratory disease, including if there will be policies in the future or not.

There were no significant differences in the acceptability levels for each measure when we stratified them by COVID-19 severity. However, there were statistically meaningful differences in the acceptability level when the smoking history categories were compared. Seven out of 13 (54%) respiratory virus infection control measures were less supported by current smokers, with an acceptability level of <70%. Only two measures were less acceptable (<70%) by participants in all smoking categories, including social distancing in an outdoor public space and wearing a face covering outdoors. Figure 3 demonstrates the distribution of the acceptability level among participants who had never smoked, current smokers, and former smokers. People with chronic lung disease strongly supported 62% of the infection control measures, and indicated that they would continue to take some steps to reduce their future risk of exacerbation. In addition, they supported 77% of the measures being implemented as mandatory policies in the future. Figure 4 shows the acceptability of respiratory virus infection control interventions in people with chronic respiratory disease, including whether they believe there should be future policies on the intervention method.

## 4. Discussion

The findings of this community survey of 2057 people indicate that many respondents desire to continue using respiratory viral infection management strategies, which were originally implemented during the COVID-19 pandemic, to lower their future risk of getting an infection. There was also widespread support (defined as a ≥ 70% acceptability level) for a continuation of such measures in the future as policies for everyone at all times. Based on evidence that these nonpharmacological interventions are effective at decreasing respiratory infections, they should be endorsed and included in future guidelines for both the general public and people living with respiratory conditions.

In both pandemic and non-pandemic situations, nonpharmacological public health measures, such as hand hygiene, face coverings, and social distancing, may offer easy, low-cost, and effective means of reducing the spread and impact of acute respiratory infections [8,9]. People in the community can use respiratory infection control measures both while they are healthy to reduce viral exposure and prevent infection and when they are sick to avoid infecting others until they fully recover. In our study, a substantial number of respondents predicted that they would continue to take precautionary measures to avoid respiratory infections in the future. Better knowledge of how individuals in various situations accept public health advice on nonpharmacological interventions may assist us in identifying areas where future public health communication efforts can be implemented. Studies have found that several personal protective measures, such as hand washing and respiratory hygiene, are broadly accepted measures for avoiding respiratory illness transmission [19,20]. Moreover, face coverings have been seen as a useful and clearly visible means of respiratory infection prevention by those who perceive themselves to be at a high risk of contracting and spreading respiratory diseases [21]. In our sample, there were statistically significant differences and differences of more than 10%, between age groups regarding such measures. People over 40 years of age were more willing than other age groups to use the measures, even if there were no policies. This may be due to their perceived understanding of the benefits of such measures in reducing transmission and disease severity. Similarly, self-isolation and social distancing behaviors were considered acceptable by the survey participants, and they would continue to apply them in the future. This finding is consistent with those who reported similar acceptance by people during the SARS pandemic; this was perceived as a method of being socially responsible [22]. There was less support (acceptability level < 70%) from our sample for wearing face coverings outdoors, social distancing outdoors, avoiding seeing friends or family if they were unwell with a cold or flu, and hand sanitizer being widely available to clean one’s hands. This could be due to several reasons previously reported in the literature. First, such measures have the potential to attract social stigma (such as being fastidious or obsessive) and cause embarrassment or discrimination [19,23,24]. Second, there is a negative impact on individuals that includes the reported physical discomfort associated with mask use as well as the perceived discomfort and impracticality associated with hand and respiratory hygiene [23,25]. Third, the practice of personal distancing was deemed inappropriate within homes and certain cultural groups because it might impede the social connections that were seen as vital for a person’s social and cultural survival [22,26]. Concerns about self-protection and personal distancing might be overcome by the perceived necessity or desire to care for sick (isolated) near and dear ones [27].

Another important feature of our research is the evaluation of community acceptability of whether respiratory infection control measures should be mandated as policies that apply to everyone at all times in the future. Of the respiratory virus infection control measures, 85% (11/13) received great support for continuation as a regulation for everyone in the future. There were variations in the level of acceptance when stratified by age groups. Those above the age of 60 were less likely than other age groups to support the availability of hand sanitizers to clean their hands. Young individuals, on the other hand, were less motivated to avoid visiting friends and relatives if they had a cold or flu. Even though there was no statistical difference across groups, only those older than 60 were in favor of social distancing in public spaces (acceptability level: 87%). Face coverings outdoors were less endorsed by all age groups. It is possible that the observed differences in acceptability levels across different age groups were influenced by personal and cultural attitudes regarding infection transmission [4].

We found smoking history to be associated with different acceptability levels among the measures. Despite smokers having an increased susceptibility to infections [28], current smokers were less supportive for seven out of 13 (54%) respiratory virus infection control measures to be implemented as policies for everyone at all times. These measures include avoiding smoking in indoor and outdoor areas, wearing a face covering on public transportation or in indoor public places, hand sanitizer being widely available to clean one’s hands, avoiding sick friends or family, social distancing in an outdoor public space, and wearing a face covering outdoors. These findings indicate that current smokers are less cautious about adopting such interventions. This could be explained by personal beliefs and the perceived vulnerability to respiratory infection among smokers [4]. In addition, most people with chronic lung disease believed they would continue their efforts to lower their risk of exacerbation by taking infection control measures in the future. In Saudi Arabia, the prevalence and incidence of COPD have steadily risen, with an estimated 434,560.64 individuals having the disease in 2019 [29]. Their support for future policy initiatives was also strong, as shown by their support for 77% of the proposed measures. This supports an earlier study by Hurst et al. (2021), which showed that a significant number of people living with chronic lung disease believed that anti-infection measures should be applied to the wider community as well as to themselves beyond the COIVD-19 pandemic, particularly during the flu season [18]. For the future implementation of such measures among smokers and people living with chronic lung disease, special consideration should be paid to the benefits and potential harms of these measures, including cost and psychological harm [18,30].

Our work has both strengths and weaknesses. The survey was conducted online, but a paper-based survey was used for those who lacked digital literacy. The sample size was adequate, although it was a convenience sample. We were the first to explore the acceptability of respiratory virus infection control measures beyond COIVD-19 in the Saudi Arabian general community, and our findings can be used and compared internationally to see how different communities accept such measures. However, regarding masks only, long-term mask use by the general public might have some negative effects in a variety of areas including the psychological, social, and physical level [31]. In addition, face masks have been shown to alter emotional inferences and social judgments [32]. As a result, an extra caution should be taken when wearing masks in order to avoid the associated negative effects, while boosting the positive effects such as the associated reduction in influenza activity [33].

This paper has important clinical and research implications. It highlights substantial support for several respiratory virus infection control interventions, which indicates a high level of tolerability for those individuals surveyed despite the high availability of vaccinations. However, nonpharmaceutical public health strategies are more likely to be adopted if common public views and concerns regarding the need, effectiveness, acceptability, and practicality of nonpharmaceutical respiratory infection management are addressed. Public health messaging should target smokers to increase their awareness of the importance of such measures in controlling infections. It is crucial to optimize the behavior-change interventions needed to maximize the benefits of continuing respiratory virus infection control strategies. Finally, these nonpharmacological measures should be recommended and included in future recommendations for both the general population and those with respiratory disorders, based on the evidence that they reduce respiratory infection [18,34,35]. Future research should explore the long-term effects of using these measures on the overall health status of the public and people with comorbidities, including the quality of life, hospitalization, and mental health.

## 5. Conclusions

The Saudi Arabian community is in favor of nonpharmacological respiratory infection control measures that lower the likelihood of infections. Public health campaigns should target smokers to spread awareness of the value of these measures in reducing infections. Based on this research, these nonpharmacological approaches should be proposed and incorporated into future recommendations for both the general population and individuals suffering from respiratory problems.

## Figures and Tables

**Table 1:** Characteristics of the respondents.

Characteristics	N (%) or Median IQR
Gender	
Male	571 (28%)
Female	1486 (72%)
Age	
18–30 years	933 (45.4%)
31–40 years	483 (23.5%)
41–50 years	448 (21.8%)
51–60 years	170 (8.3%)
>60 years	23 (1.1%)
Body Mass Index (BMI)	26 (22–30)
Education Level	
No formal schooling	12 (0.6%)
Elementary school	14 (0.7%)
Intermediate school	65 (3.2%)
High school	511 (24.8%)
Diploma	240 (11.7%)
Bachelor’s	1114 (54.2%)
Master’s	70 (3.4%)
Ph.D.	31 (1.5%)
Region	
Central	545 (26.5%)
Eastern	372 (18.1%)
Northern	183 (8.9%)
Southern	122 (5.9%)
Western	835 (40.6%)
Where do you live?	
Rural area	245 (11.5%)
Urban area	1812 (88.1%)
Comorbidities	
Chronic lung disease	72 (3.5%)
Cardiac disease	22 (1.1%)
High blood pressure	128 (6.2%)
Kidney disease	9 (0.4%)
Diabetes	123 (6%)
Stomach disease	81 (3.9%)
Liver disease	8 (0.4%)
Anemia or other blood disease	85 (4.1%)
Cancer	7 (0.3%)
Depression	70 (3.4%)
Osteoarthritis	70 (3.4%)
Smoking history	
Never smoked	1697 (82.5%)
Current smoker	259 (12.5%)
Former smoker	101 (4.9%)
Pack-years	10 (5–20)
If a smoker, have you attempted to quit during the COVID-19 pandemic?	174/259 (67%)
Have you been infected with COVID-19?	
Yes	889 (43.2%)
Severity of COVID-19 infection	
Mild	250 (28%)
Moderate	501 (56%)
Severe	138 (16%)
Did any of your close family members get infected with COVID-19?	
Yes	1716 (83.4%)
No	314 (16.6%)
Have you lost any loved ones because of the COVID-19 infection?	
Yes	572 (27.8%)
No	1485 (72.2%)
Have you been vaccinated against COVID-19?	
Yes	2028 (98%)
First dose (Incomplete vaccination)	19 (1%)
Second dose	807 (40%)
Booster dose	1202 (59%)

**Table 2:** Acceptability of respiratory virus infection control interventions. Data are percentages of participants answering yes (n = 2057). BOLD indicates a ≥ 70% acceptability level.

Respiratory Infection Control Measure	Thinking about the Future, after Restrictions Have Been Eased and People Get Vaccinated against COVID-19, Which One Would You Do (Even If It Was Not Policy); N (%)	Which of the Following Do You Think Should Continue in the Future as a Policy For Everyone at All Times? N (%)
Washing hands more often	1910 (93%)	1923 (93%)
Wearing masks in hospitals (both HCP and patients)	1886 (92%)	1894 (92%)
Self-isolating if they were in contact with someone who was infected with COVID-19	1878 (91%)	1889 (92%)
Taking vaccinations as recommended by the ministry of Health	1793 (87%)	1838 (89%)
Avoiding smoking in indoor and outdoor areas	1759 (86%)	1814 (88%)
Keeping more of a distance from others when in an indoor public space	1722 (84%)	1806 (88%)
Avoiding busy public spaces	1689 (82%)	1838 (89%)
Wearing a face covering on public transportation	1509 (73%)	1596 (78%)
Wearing a face covering in indoor public places	1438 (70%)	1554 (76%)
Having hand sanitizer be widely available to clean hands	1382 (67%)	1565 (76%)
Avoiding seeing friends or family if they are unwell with a cold or flu	1304 (63%)	1493 (73%)
Keeping more of a distance from others when in an outdoor public space	1243 (60%)	1353 (66%)
Wearing a face covering outdoors	793 (39%)	928 (45%)

## Data Availability

The data presented in this study are available upon request from the corresponding author.
